# Prolonged survival time of *Daphnia magna* exposed to polylactic acid breakdown nanoplastics

**DOI:** 10.1371/journal.pone.0290748

**Published:** 2023-09-05

**Authors:** Egle Kelpsiene, Melinda Rydberg, Mikael T. Ekvall, Martin Lundqvist, Tommy Cedervall

**Affiliations:** 1 Department of Biochemistry and Structural Biology, Lund University, Lund University, Lund, Sweden; 2 NanoLund, Lund University, Lund, Sweden; 3 Department of Biology, Ecology Building, Aquatic Ecology Unit, Lund University, Lund, Sweden; Annamalai University, INDIA

## Abstract

Polylactic acid nanoparticles (PLA NPs) according to food and drug administration are biodegradable and biocompatible polymers that have received a lot of attention due to their natural degradation mechanism. Although there is already available information concerning the effects of PLA microplastic to aquatic organisms, the knowledge about PLA NPs is still vague. In the present study, we analyzed the chemical composition of engineered PLA NPs, daily used PLA items and their breakdown products. We show that PLA breakdown products are oxidized and may contain aldehydes and/or ketones. The breakdown produces nanosized particles, nanoplastics, and possibly other small molecules as lactide or cyclic oligomers. Further, we show that all PLA breakdown nanoplastics extended the survival rate in *Daphnia magna* in an acute toxicity assay, however, only PLA plastic cup breakdown nanoplastics showed a significant difference compared to a control group.

## Introduction

Plastics are polymers with multiple applications and have an important role in our daily life. It has been estimated that between 1950 and 2020, global plastic production increased from 1.5 million metric tons to 367 million metric tons per year [1]. The increased production of plastics results in a growing amount of plastic material misplaced in the environment. Approximately between 60 to almost 100 million tons are mismanaged and ∼90% of it ends up in waterways and potentially reaches the oceans [2]. Therefore, the effects of plastic pollution on the aquatic environment have attracted both societal and scientific concerns [3–5].

Micro- (< 5 mm) and nano-sized(< 1 μm or <100 nm) particles can be either produced intentionally or degrade to smaller fragments under natural [6–9] or laboratory conditions [10–15]. Regardless of the particle preparation or size, their waste and breakdown products will eventually reach the natural environment and become a potential threat to both aquatic fauna and flora [16–19].

Main advantages of plastics are that they are light in weight, inert, long lasting and are cheap to produce. However, their high molecular weight, complex three-dimensional structure and hydrophobic nature prevent their degradation which can lead to accumulation of enormous quantities in the natural environment [20]. Taking this into account, biodegradable or biocompatible plastics, such as polylactic acid (PLA)-based polymers, may be good candidates to replace non-biodegradable plastics [21].

PLA is classified as an aliphatic polyester because of the ester bonds that connect the monomer units [22]. PLA has received a lot of attention in the biomedicine field [23–25] due to its natural degradation *in situ* through hydrolysis mechanism, where water molecules break the ester bonds which create a polymer backbone [22]. Degradation products are composed of lactic acid and its short oligomers. These products are identified and metabolized by the body itself, which gives PLA an intrinsic biocompatibility that dampens the attainment of critical immune responses [22]. It is worth mentioning that PLA can be combined with biodegradable and non-biodegradable polymers, such as polyethylene (PE), polypropylene (PP), chitosan, polystyrene (PS), polyethylene terephthalate (PET) or polycarbonates [26]. Moreover, the addition of carbon nanotubes, ceramic nanoparticles, natural fibers, or cellulose while making composite materials [27–29]. This suggests that tracers of other polymers can be found in the PLA breakdown products.

Nanoparticles behavior and toxicity mainly depends on its size, shape and, surface charge [30,31]. Typical unwanted effects are related to oxidative stress, apoptosis, cytokine activation, loss of mitochondrial and lysosomal stability, genotoxic effects or DNA damage [32]. PLA nanoparticles (NPs) can provide additional adverse effects through their degradation products. It has been shown that engineered PLA NPs with sizes of 63 nm and 66 nm can be generally tolerated by human lung epithelial A549 cells (HLE A549), with no cytotoxicity and no secretion of pro-inflammatory mediators [33]. However, PLA NPs of the same sizes induced changes to the proteome of HLE A549 cells [33]. Additionally, *in vitro* study showed that ∼75 nm PLA NPs exhibited higher toxicity in RAW 264.7 macrophage cell line in comparison to larger ∼160 nm PLA NPs [34]. Additionally, smaller-sized PLA NPs (∼75 nm) induced a higher dose-dependent reactive oxygen species (ROS) production compared to larger-size (∼160 nm) PLA NPs [34].

Zimmermann and co-authors [35] showed that reproduction output was reduced significantly in the freshwater filter feeder *Daphnia magna* after exposure to 40 μm PLA microplastics (MPs) at 500 mg/L compared to a control group. Additionally, mortality of *D*. *magna* increased after exposure to 40 μm PLA MPs in a concentration-dependent manner (from 10 mg/L to 500 mg/L) to 60%. Authors also showed that *D*. *magna* has significantly lower mean body length at 500 mg/L of 40 μm PLA MPs [35]. Furthermore, it has been observed that ultraviolet radiation degraded products of PLA MPs (∼11 μm) at 25 mg/L elevated ROS levels, mitochondrial damage and apoptosis in zebrafish larvae [36].

Manufactured plastic NPs have been shown to be different compared to breakdown nanoplastics [11]. Therefore, in the present study, we characterized the chemical composition of pure engineered PLA NPs, bulk material of different PLA items that are available in supermarkets and used in daily life and their breakdown products. Additionally, we elucidated the toxicity of both engineered PLA NPs and PLA breakdown particles by using the well-studied model organism *D*. *magna*.

## Materials and methods

### Preparation of PLA nanoplastics

Manufactured PLA NPs (250 nm) were purchased from CD Bioparticles (www.cd-bioparticles.com). Before the experiments, PLA NPs were diluted to 10 mg/L and dialyzed in a Standard RC Tubing, Dialysis Membrane (MWCO: 3.5 kD) for 72 h at 4 ˚C in 10 L of MiliQ water. The water was changed after 4 h the first day and once a day on the following days.

Different types of PLA items, such as soup cup lids, 3D printer filaments, and plastic cups, were bought in supermarkets in Lund, Sweden. The PLA breakdown nanoplastics were prepared in the similar manner as published previously for PS and high-density polyethylene (HDPE) [10,11]. Briefly, 2 g of PLA product was cut into small pieces (ca. 1x1 cm) into a glass beaker. The beaker was then filled with 200 mL tap water and the blender was turned on at maximum speed for 2 minutes. A 50 mL syringe was used to remove 100 mL of the water, which was filtered through a 0.8 μm cellulose acetate syringe-filter (Whatman, GE) into a glass bottle storage container. If more particle solution was needed, the larger beaker was filled with another 100 mL tap water, and the blending process and filtering was repeated. The same exact breakdown procedure was repeated using MiliQ water, as it was used for nanoplastics characterization.

### Characterization of PLA nanoplastics

The number concentration and size of both dialyzed and non-dialyzed PLA NPs, and PLA breakdown nanoplastics were analyzed by nanoparticle tracking analysis (NTA) NanoSight LM10 (Amesbury, UK) on the same day as particles were prepared (i.e., day 0) and 6 days after breakdown procedure to evaluate if any aggregation of particles occurred. Additionally, the size of PLA breakdown nanoplastics were measured in Mili-Q water on day 0 of the breakdown procedure by using dynamic light scattering (DLS) on a Zetasizer Nano S (Malvern instruments, Worcestershire, UK). The zeta potential using Zetasizer Ultra or Zetasizer Nano ZS (Malvern Instruments, Worcestershire, UK) was used to measure the stability of nanoplastics. Before the zeta potential measurements, PLA nanoplastics were concentrated using a VivaFlow (VIVAFLOW 50, Sartorius) to improve the data collection. Measurements were repeated three times and data is presented as an average value.

Fourier transformed infrared spectrometry (FTIR) was performed on a Spectrum Two (PerkinElmer) using the software PerkinElmer Spectrum IR version 10.7.2 in the spectra range of 4000–450 nm. The samples for the PLA bulk material were added directly on the crystal, whereas liquid samples (5 μL) added on the crystal were left to evaporate before measurements. The acquired spectra were compared to the spectra in the software library.

PLA NPs and PLA soup lid nanoplastics were additionally analyzed by transmission electron microscopy (TEM). Briefly, 2 μL of the sample was added to a pioloform-coated single slot grid (Ted Pella, Cu, Pelco Slot Grids, USA), and left to air dry overnight. The samples were then inserted into a JEOL JEM-1400 PLUS TEM operated at 100 kV (JEOL Ltd., Japan), where micrographs were obtained using TEM Centre for JEM1400 Plus software.

### Study organism

The freshwater filter feeder *D*. *magna* was used in the present study as a model organism. The original culture originates from lake Bysjön (55° 40′ 31.3″ N, 13° 32′ 41.9″E) and has been kept under controlled laboratory conditions for several generations. The *D*. *magna* cultures were fed *ad libitum* 2–3 times a week with a culture of the green algae *Scenedesmus* sp. All cultures were maintained at 18°C at 8:16 h light/dark photoperiod.

### Acute toxicity tests

Before the toxicity test, PLA products were broken in the tap water which was first filtered through 0.2 μm syringe filter to remove any bacteria that might come with the tap water. First, we evaluated the effects of PLA broken down nanoplastics on *D*. *magna*. There were four experimental groups: control group, soup lid nanoplastics, 3D printer filament nanoplastics, and plastic cup nanoplastics. The particle solution was aliquoted to 50 mL Falcon tubes with a total volume of 25 mL. One *D*. *magna* (2–3 days old) that came from the same culture was randomly distributed into each tube (in total 15 replicates per each treatment).

Secondly, we investigated the effects of engineered PLA NPs on *D*. *magna* individuals. One *D*. *magna* (2–3 days old) were randomly assigned to 50 mL Falcon tubes (in total 10 replicates for each treatment) with a total volume of 25 mL. *D*. *magna* were exposed to dialyzed PLA NPs (with different dilution factors, 1:1, 1:10, or 1:100), non-dialyzed PLA NPs (with different dilution factors, 1:1, 1:10, or 1:100) or water only (control). The highest concentration (1:1) for dialyzed or non-dialyzed PLA NPs was 10 mg/L. During all toxicity tests, individuals were checked once a day until all of them were immobilized, and during the exposure period *D*. *magna* were not fed.

### Statistical analysis

Kaplan Meier survival curves analysis were performed using the statistical computing software GraphPad Prism version 9.3.1 (471) for Windows (GraphPad Software, Inc., ww.graphpad.com). The analysis performed were the Log Rank (Mantel-Cox) test and the Gehan-Breslow-Wilcoxon test.

## Results and discussion

### Characterization of PLA nanoplastics

**Fig 1:**
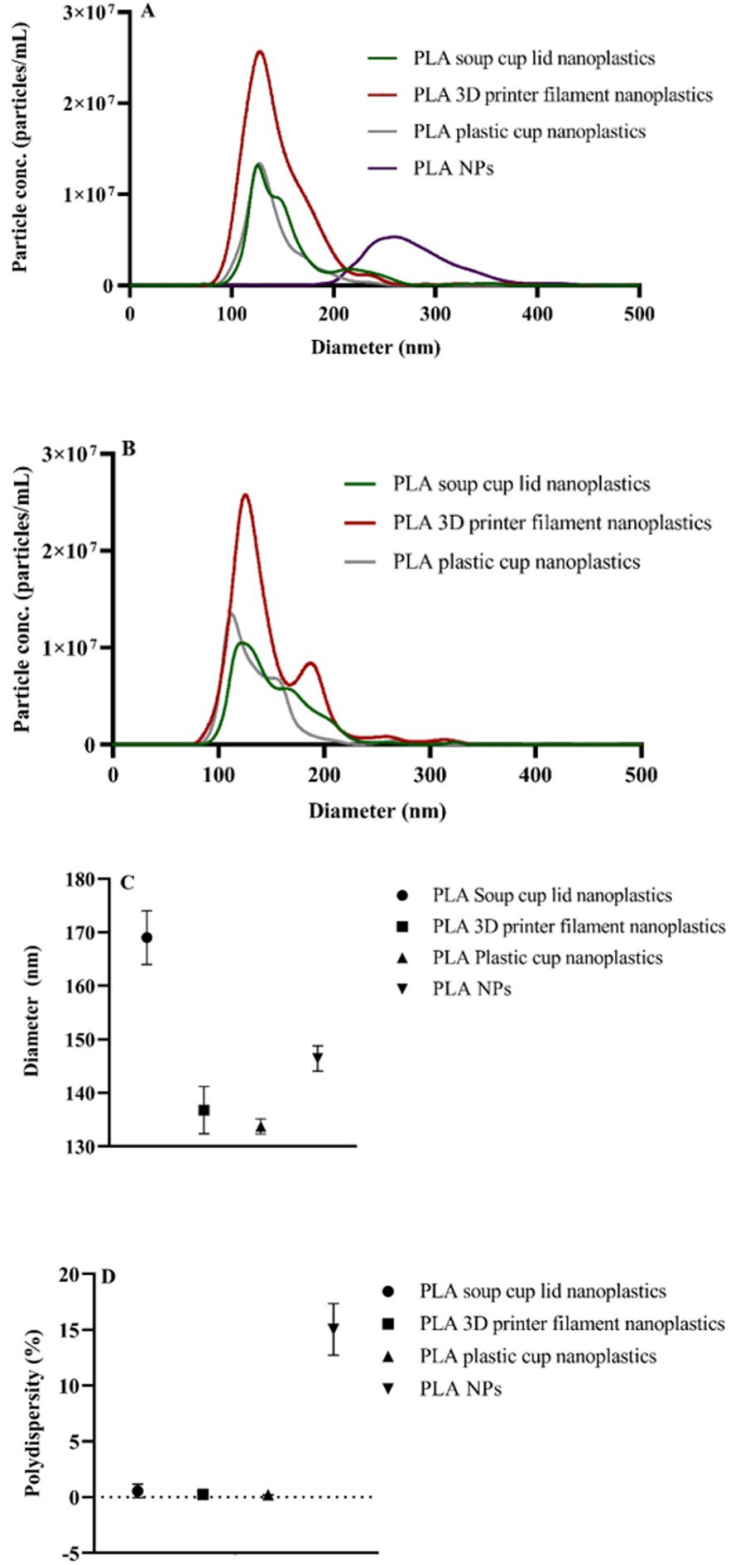
The distribution of PLA NPs and breakdown fractions determined by NTA (A-B) and DLS (C-D). Each sample is an average from five recordings. Measurements for NTA were performed the same day (day 0) of the breakdown procedure (A) and 6 days after the breakdown procedure (B). PLA NPs and breakdown products sizes (C) and polydispersity (D) were measured in triplicates by DLS on day 0.

**Fig 2:**
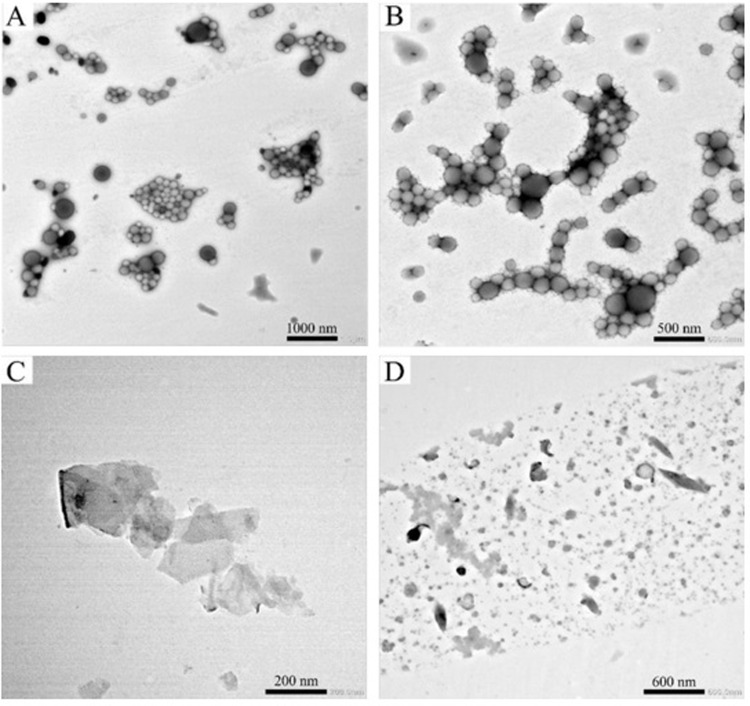
TEM images of PLA NPs (A-B) and PLA soup lid breakdown nanoplastics (C-D). TEM images showed that majority of the particles were around 100–150 nm.

NTA measurements showed that the mean size was ∼170 nm for all PLA breakdown nanoplastics, and ∼270 nm for PLA NPs (Fig 1A). To ensure that nanoplastics did not aggregate, NTA measurements were repeated 6 days after the breakdown procedure, showing that PLA nanoplastics´ dispersions were stable (Fig 1B). DLS measurements showed that sizes of PLA nanoplastics were between ∼130 and 170 nm (Fig 1C), with a low polydispersity for PLA nanoplastics and higher polydispersity for PLA NPs (Fig 1D). Zeta potential measurements showed that all PLA NPs and PLA breakdown nanoplastics had negative surface charge (-22.13, -9.59 and -14.94 for PLA soup lid breakdown nanoplastics, PLA plastic cup breakdown nanoplastics and PLA NPs, respectively). Interestingly, PLA 3D printer filament nanoplastics had both negative and positive surface charges (-44.41 and 42.67). Sizes of PLA NPs were additionally measured by NTA after dialysis to ensure that dialysis did not induce particle aggregation (S1 Fig in S1 File). PLA NPs and PLA soup lid nanoplastics were further analyzed by TEM, showing that PLA NPs were round-shaped single particles as well as mainly dendritic-shaped aggregates (Fig 2A and 2B), whereas PLA soup lid nanoplastics were irregular in both shapes and size (Fig 2C and 2D).

The chemical signature of the pure engineered 250 nm PLA NPs was determined by FTIR. Obtained spectra had peaks at ∼2930 cm^-1^ between ∼1750 cm^-1^ and 1090 cm^-1 ^region (S2 Fig in S1 File). Similarly, peaks of PLA particles were observed at the same wavelengths in other studies [37–39], suggesting that our PLA NPs were pure and with no additives. The spectrum of PLA NPs was used as a reference spectrum to compare the spectra of different PLA products and chemical changes during the breakdown procedure.

**Fig 3:**
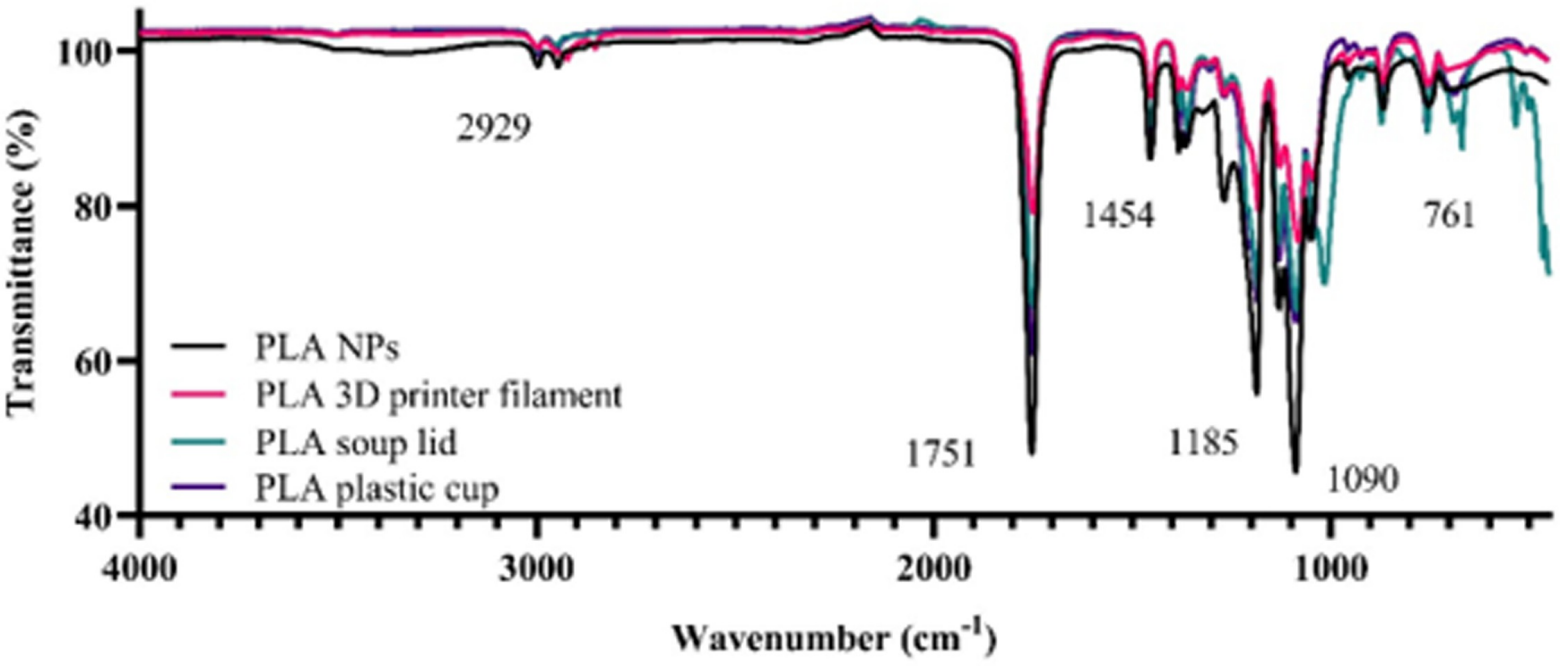
FTIR spectra of the different PLA bulk materials in comparison with engineered PLA NPs.

The spectrum of bulk material of PLA plastic cup, PLA soup lid and PLA 3D printer filament showed a similarity of 92%, 90% and 86%, respectively, to spectrum obtained for pure engineered 250 nm PLA NPs (Fig 3). In addition, PLA products had peaks at ∼2930 cm^-1^ and between ∼1750 cm^-1^ and 1090 cm^-1 ^region, and some less pronounced peaks around ∼750 cm^-1^ region compared to a spectrum obtained for PLA NPs (Fig 3).

**Fig 4:**
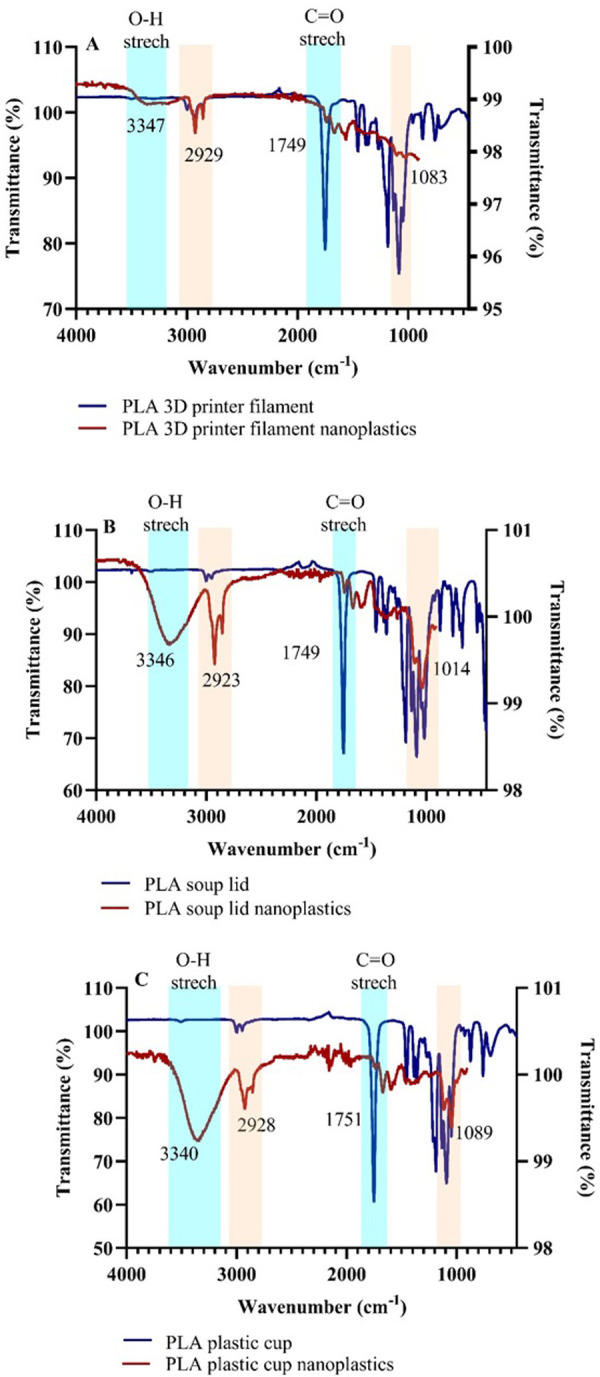
FTIR spectra of different PLA bulk material (blue line, left Y axis) and their breakdown products (red line, right Y axis). Light orange color indicates peaks that are indicated similarities between bulk material and its breakdown products, whereas light blue color indicates oxidation that occurred during the breakdown process in PLA nanoplastics.

The spectrum obtained for PLA bulk materials have sharp peaks between ∼1000 cm^-1^ and ∼2000 cm^-1^ due to triple bonds (e.g., C≡C or C≡N), which within the breakdown procedure start to be less pronounced (Fig 4). Broad peaks between ∼3500 and ∼3000 cm^-1^ region and smaller peaks at ∼1700 cm^-1^ for PLA breakdown nanoplastics might be due to of O–H or C = O stretching vibrations, of ethers or carbonates [10,40]. This implies that oxidation, as well as aldehydes and/or ketones were formed during the degradation of PLA products [41], additionally breakdown products include lactide or cyclic oligomer both by ester interchange and by chain homolysis route of PLA [41,42].

### Acute toxicity tests

**Fig 5:**
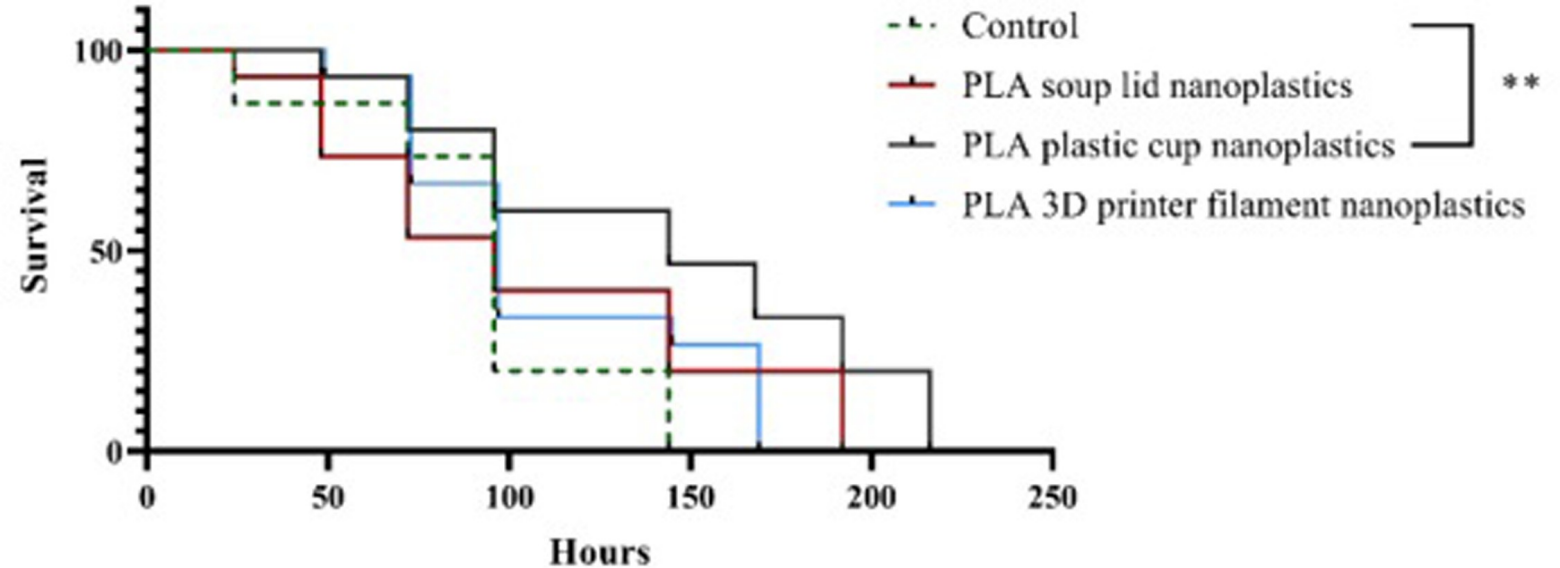
Survival of *Daphnia magna* exposed to different PLA breakdown nanoplastics. The curve of PLA 3D printer filament nanoplastics was nudged on Y axis by 1.00 data units for clearer vision. In total there were 15 replicates for each treatment. Asterisk indicates significant difference among the treatments estimated over the study period, **p<0.01.

First, we investigated the effects of PLA breakdown nanoplastics on *D*. *magna*. The data the survival of *D*. *magna* was significantly extended after exposure to PLA plastic cup nanoplastics (χ ^2^_(1)_ = 4.93, p < 0.01, Fig 5) in comparison with a control group, whereas other PLA breakdown nanoplastics did not have any significant effects towards *D*. *magna* survival.

**Fig 6:**
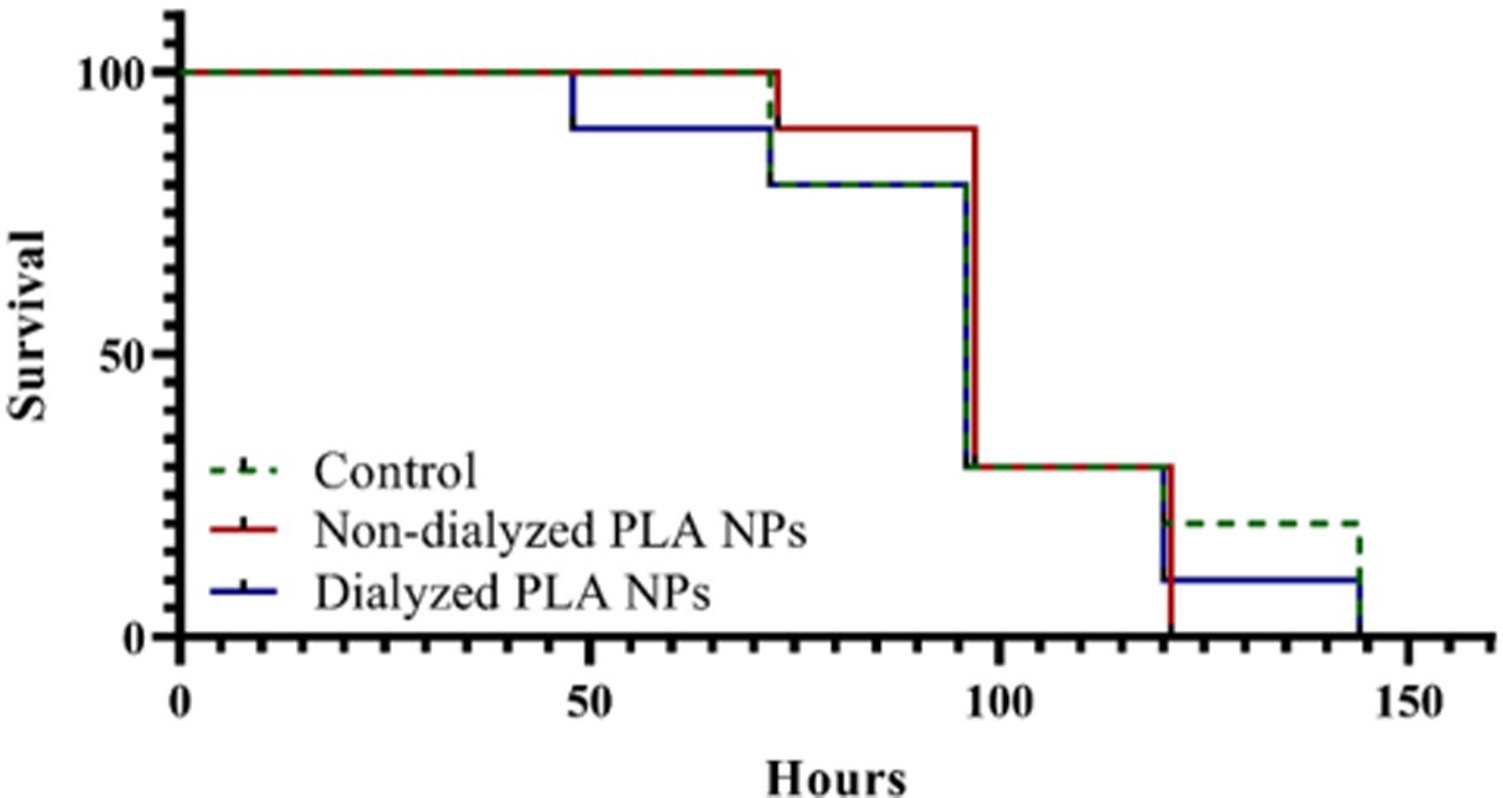
Survival of *Daphnia magna* exposed to dialyzed and non-dialyzed 250 nm PLA NPs. The curve of non-dialyzed PLA NPs was nudged on Y axis by 1.00 data units for clearer vision. There were 10 replicates for each treatment.

Secondly, we wanted to investigate if engineered PLA NPs would have the same or similar effect as PLA breakdown products. Additionally, as previously, it has been highlighted by several authors the importance of washing nanoparticles to remove additives before performing toxicity studies [43,44], therefore here, we compared dialyzed versus non-dialyzed PLA NPs with different dilution factors (S3 Fig in S1 File). The data shows that PLA NPs did not extend or affect the survival for *D*. *magna* after exposure to 10 mg/L either dialyzed or non-dialyzed PLA NPs in comparison with control group (χ ^2^_(1)_ = 0.03, p > 0.99, overall analysis for all treatments, Fig 6).

In the present study, we have observed that *D*. *magna* survival was significantly extended after exposure to PLA plastic cup breakdown nanoplastics. Even though other PLA breakdown nanoplastics used in the study did not have significant effects on *D*. *magna* survival, however there was a trend for prolonged survival after exposure to PLA soup lid nanoplastics and PLA 3D printer filament nanoplastics. Ekvall and co-authors [10] showed a similar tendency, as exposure of HDPE to *D*. *magna* prolonged the survival in a life-time (∼100 days) experiment, after removal of small molecules, smaller than∼10 kDa. The extended survival for exposed individuals can be potentially explained by the growth of bacterial communities and interaction with breakdown nanoplastics. The fact that *Daphnia* individuals were not fed with algae during the exposure, suggest that bacteria might come from *Daphnia* itself. Cooper and Cressler [45] analyzed *D*. *magna* microbiota and found out that *Pedobacter*, *Flavobacterium*, *Polaromonas*, *Limnohabitans* and unclassified Burkholderiaceae were most abundant bacteria genera among other 18 genera present in *D*. *magna* samples. Similar results were obtained by other authors showing that *D*. *magna* microbiome was dominated by Proteobacteria and Bacteroidetes phylum [46–49]. A possible advantage from bacteria, for example *Pedobacter*, *Limnohabitans* or *Polaromons*, that they are able to provide amino acids and/or biosynthesize vitamins [45], which can serve fitness benefits to the host [50–52].

Bacterial communities can interact with NPs, including nanoplastics, and form a slimy and slippery layer called biofilm [53,54]. Biofilm is an aggregate of microorganisms living in a self-produced matrix of extracellular polymeric substances (EPS) which can adhere to both biotic or abiotic surfaces [54–56]. Additionally, plastics leach dissolved organic matter during plastic degradation [57,58]. This leachate coming from the plastic can provide energy for bacterial growth [59,60]. It has been shown that bacterial growth was 1.72 times higher with plastic leachate from plastic bags made of low-density PE due to the added carbon, which was more accessible than natural organic matter [61]. The bacteria then can be fed by *D*. *magna* and used as nutrients [62], which can explain the prolonged survival observed in the present study. Biofilms on the aggregates can improve animal nutrition, especially when there is low food availability [63]. Amariei and co-authors [64] showed that *D*. *magna* decreased mortality after 14-day exposure to bio-fouled PE MPs with irregular shape and size ranging from 10 to 50 μm in contrast to pristine PE MPs. Biofilm consumption has been shown to increase the survival and growth rates for several cultivated organisms, such as tilapias *Orechromis niloticus*, whiteleg shrimp *Litopenaeus vannamei*, and fringe-lipped carp *Labeo fimbriatus* [65–67]. Microorganisms that are present in the biofilm provide with essential nutrients such as polyunsaturated fatty acid, sterols, amino acids, vitamins and pigment that help to improve development of organisms [68]. Additionally, biofilms have been observed on surfaces of different type of plastics, such as PLA, PP, PE, polyvinyl chloride, HDPE, and low-density PE [69–71].

So far, micro- and nano-sized particles have been mainly studied in terms on their adverse effects to organisms due to the particle size and/or surface charge. However, the phenomenon observed in the present study, where daphnids extended the survival after exposure to different PLA breakdown nanoplastics, highlights the need to take a closer look at the microbial composition and nutrient quality of the biofilm and its interaction with nanomaterials by using different type of plastics. Further, considering that various plastic types might contain different additives, which can to some extent influence the bacterial composition, future studies should focus on analyzing biofilm bacterial composition and its interaction with both pure nanomaterials and their additives. Finally, studies should include longer incubation time and/or seasonal factors.

## Supporting information

S1 File S1 Fig. Sizes of PLA NPs were measured before and after dialysis by using NTA to ensure that particle aggregation did not occur during dialysis; S2 Fig. Spectra of engineered 250 nm PLA NPs obtained by FTIR; S3 Fig. Survival of *Daphnia magna* exposed to non-dialyzed (Fig A) dialyzed (Fig B) 250 nm PLA NPs with different dilution factors. The highest concentration (1:1) for both dialyzed and non-dialyzed PLA NPs was 10 mg/L. The experiment was performed at once, however for clearer vision survival curves for non-dialyzed and dialyzed in comparison with a control are shown separately. No statistically significances were observed among the treatments. In total there were 10 replicates for each treatment. Experiment was repeated 3 times.
(DOCX)
