# Supplementary material for: Prolonged survival time of Daphnia magna exposed to polylactic acid breakdown nanoplastics
Source: PLoS One. 2023 Sep 5;18(9):e0290748. doi: 10.1371/journal.pone.0290748 (PMC10479899; doi:10.1371/journal.pone.0290748)
Supplement: S1 File — S1 Fig. Sizes of PLA NPs were measured before and after dialysis by using NTA to ensure that particle aggregation did not occur during dialysis; S2 Fig. Spectra of engineered 250 nm PLA NPs obtained by FTIR; S3 Fig. Survival of Daphnia magna exposed to non-dialyzed (Fig A) dialyzed (Fig B) 250 nm PLA NPs with different dilution factors. The highest concentration (1:1) for both dialyzed and non-dialyzed PLA NPs was 10 mg/L. The experiment was performed at once, however for clearer vision survival curves for non-dialyzed and dialyzed in comparison with a control are shown separately. No statistically significances were observed among the treatments. In total there were 10 replicates for each treatment. Experiment was repeated 3 times. (DOCX) [file pone.0290748.s001.docx]

**Supplementary Information**

**Prolonged survival time of *Daphnia magna* exposed to polylactic acid breakdown nanoplastics**

Egle Kelpsiene ^a,b^, Melinda Rydberg ^a^, Mikael T. Ekvall ^c,b^, Martin Lundqvist ^a,b^, and Tommy Cedervall ^a,b*^

^a^ Department of Biochemistry and Structural Biology, Lund University, Lund University, P.O. Box 124, SE-221 00 Lund, Sweden

^b^ NanoLund, Lund University, Box 118, SE-221 00 Lund, Sweden

^c^ Aquatic Ecology Unit, Department of Biology, Ecology Building, Lund University, 223 62 Lund,

Sweden

*Corresponding author: [tommy.cedervall@biochemistry.lu.se](mailto:tommy.cedervall@biochemistry.lu.se)


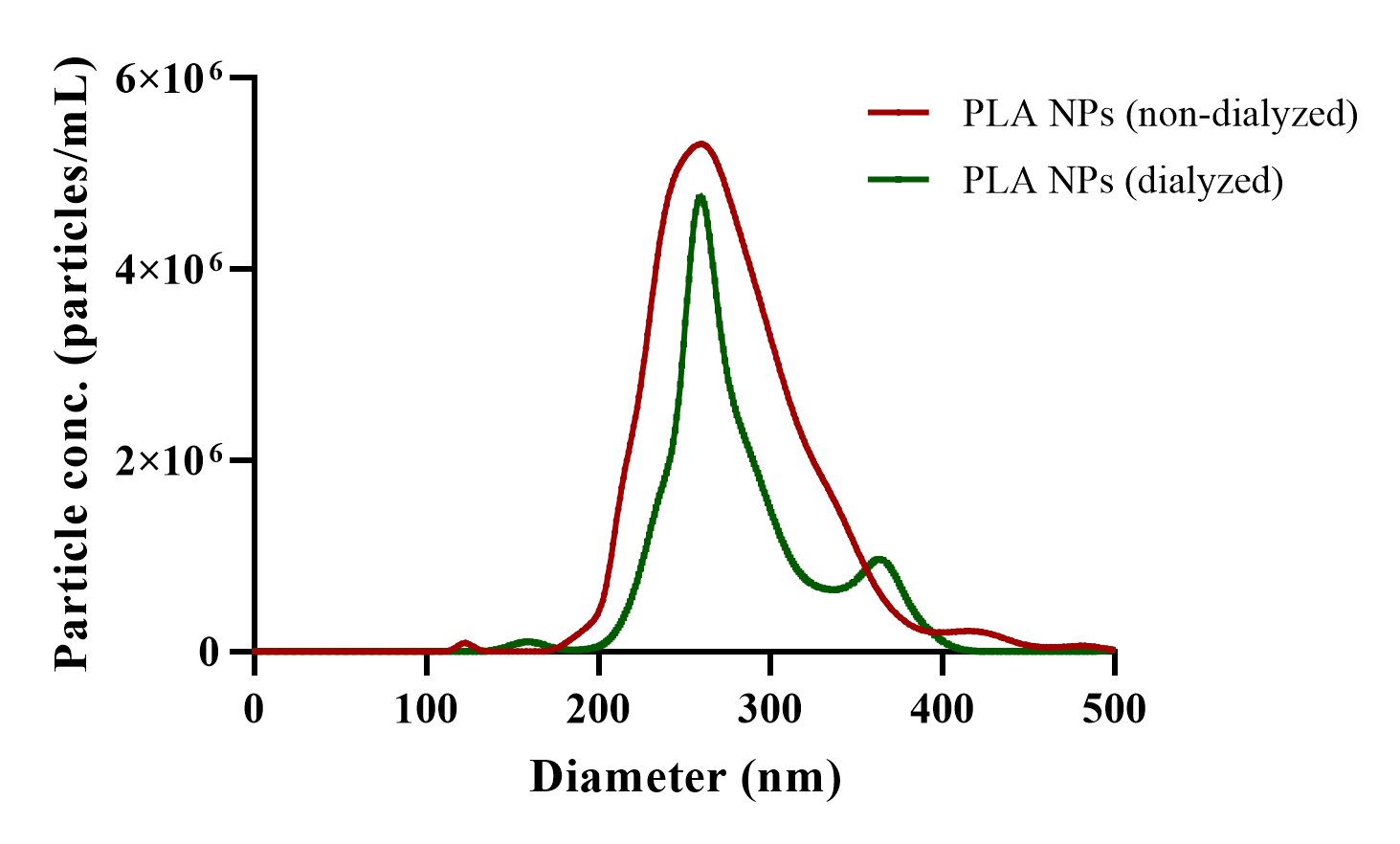


Supplementary Figure 1. Sizes of PLA NPs were measured before and after dialysis by using NTA to ensure that particle aggregation did not occur during dialysis.


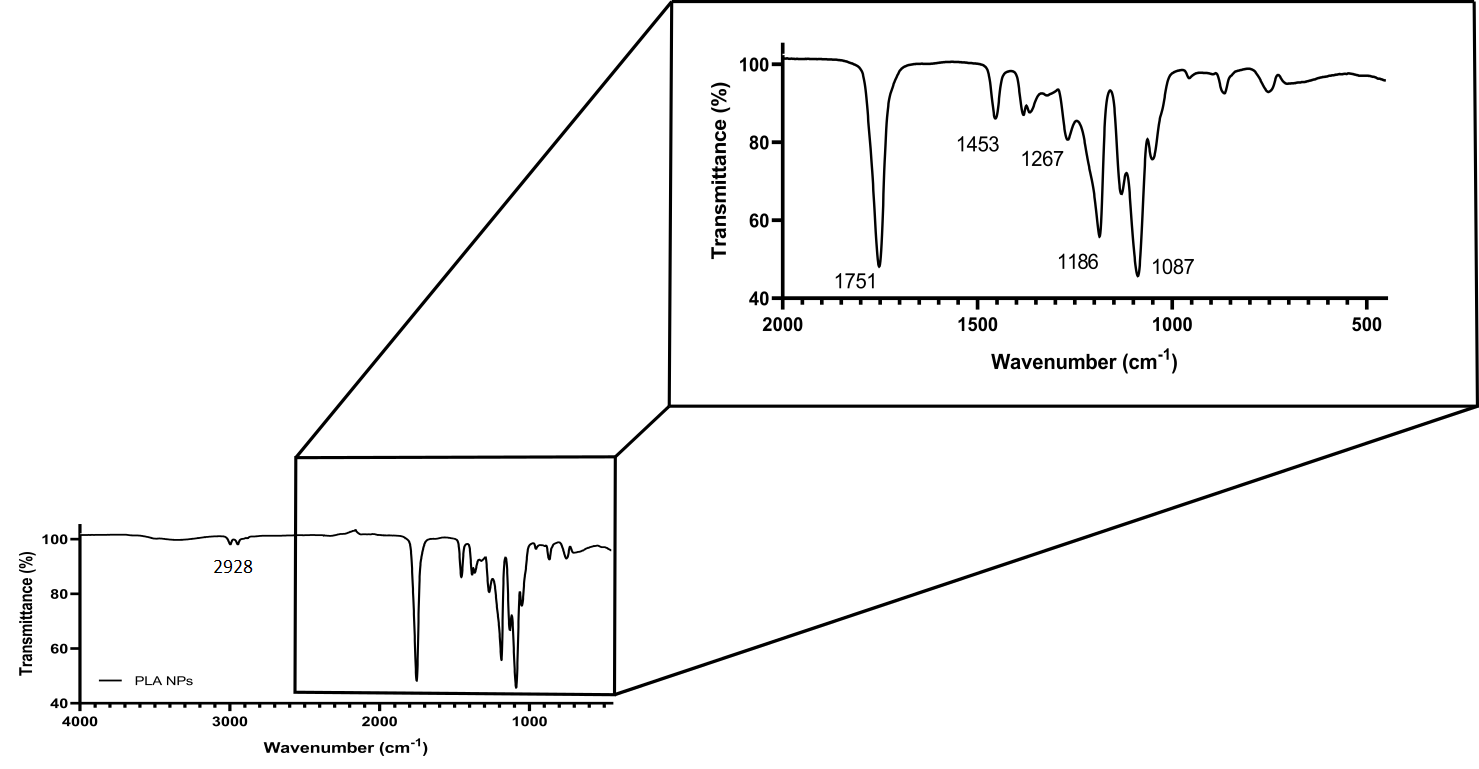


Supplementary Figure 2. Spectra of engineered 250 nm PLA NPs obtained by FTIR.


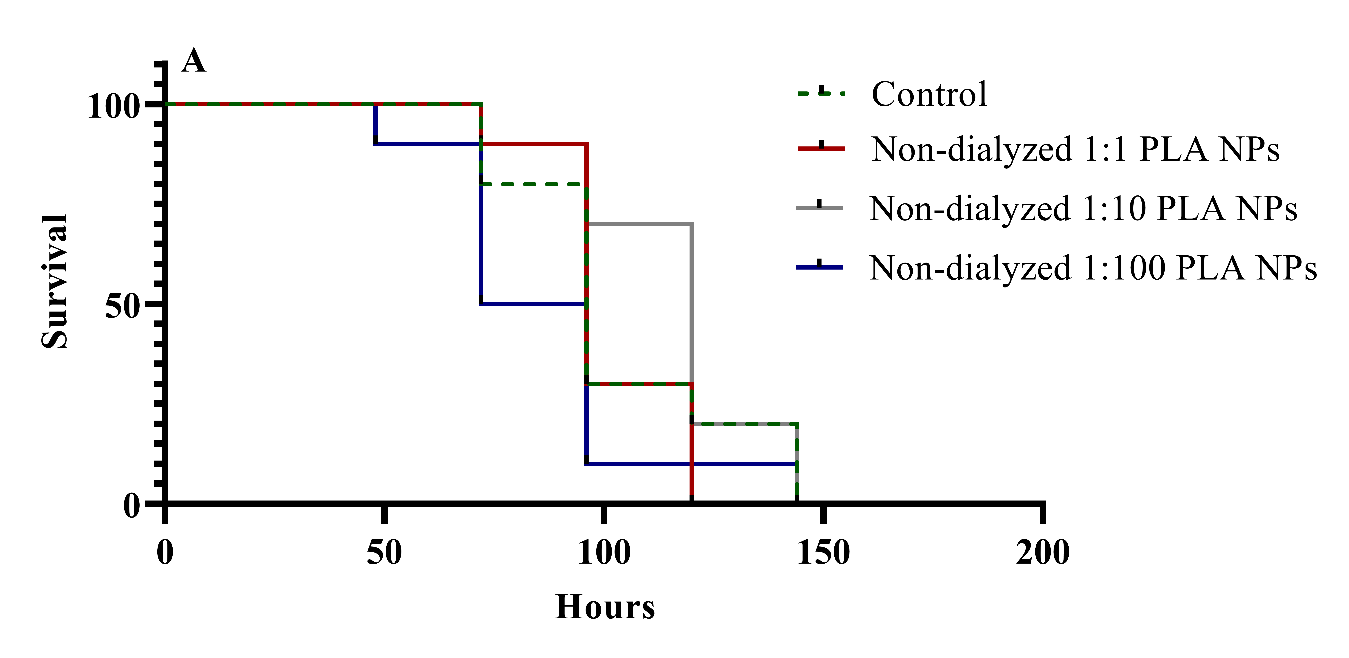


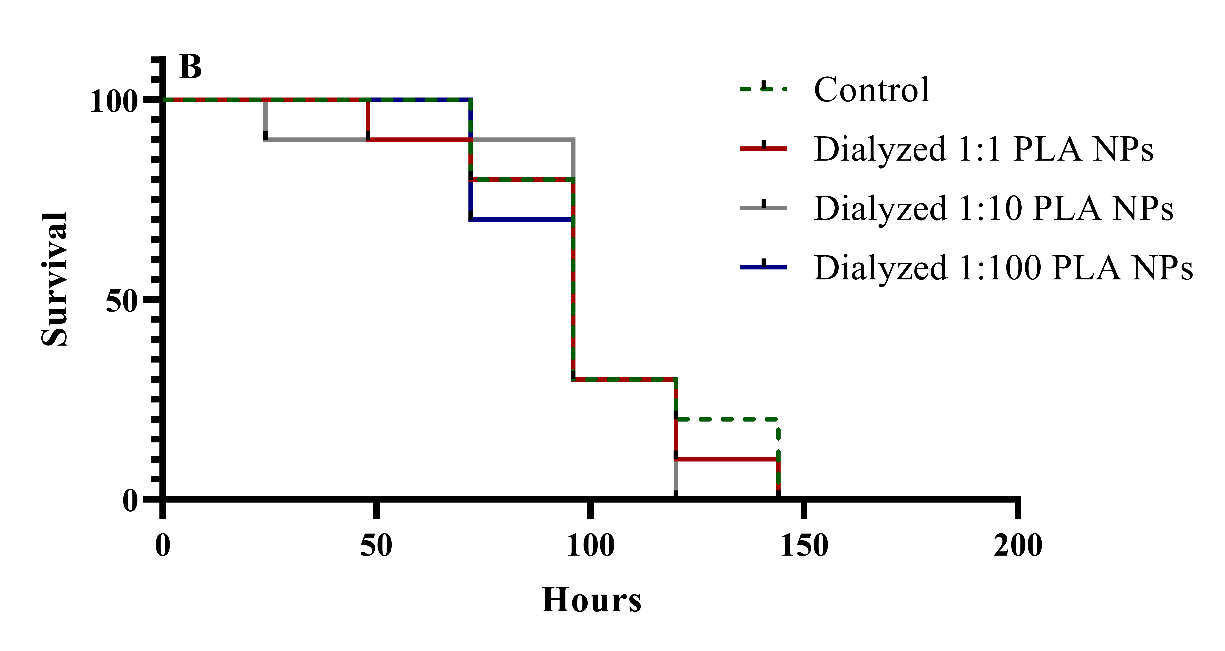


Supplementary Figure 3. Survival of *Daphnia magna* exposed to non-dialyzed (Figure A) dialyzed (Figure B) 250 nm PLA NPs with different dilution factors. The highest concentration (1:1) for both dialyzed and non-dialyzed PLA NPs was 10 mg/L. The experiment was performed at once, however for clearer vision survival curves for non-dialyzed and dialyzed in comparison with a control are shown separately. No statistically significances were observed among the treatments. In total there were 10 replicates for each treatment. Experiment was repeated 3 times.
